# Early Pharmacological Profiling of Antiproliferative Compounds by Live Cell Imaging

**DOI:** 10.3390/molecules27165261

**Published:** 2022-08-17

**Authors:** Adrián Puerta, Aday González-Bakker, Guido Santos, José M. Padrón

**Affiliations:** 1 BioLab, Instituto Universitario de Bio-Orgánica “Antonio González”, Universidad de La Laguna, Avenida Astrofísico Francisco Sánchez 2, 38206 La Laguna, Spain; 2 Departament of Biochemistry, Microbiology, Cell Biology and Genetics, Faculty of Sciences, Universidad de La Laguna, Avenida Astrofísico Francisco Sánchez s/n, 38206 La Laguna, Spain

**Keywords:** phenotypic drug discovery, cancer, natural products, live cell imaging, machine learning, antimitotic drugs, microtubule binding drugs

## Abstract

Natural products represent an excellent source of unprecedented anticancer compounds. However, the identification of the mechanism of action remains a major challenge. Several techniques and methodologies have been considered, but with limited success. In this work, we explored the combination of live cell imaging and machine learning techniques as a promising tool to depict in a fast and affordable test the mode of action of natural compounds with antiproliferative activity. To develop the model, we selected the non-small cell lung cancer cell line SW1573, which was exposed to the known antimitotic drugs paclitaxel, colchicine and vinblastine. The novelty of our methodology focuses on two main features with the highest relevance, (a) meaningful phenotypic metrics, and (b) fast Fourier transform (FFT) of the time series of the phenotypic parameters into their corresponding amplitudes and phases. The resulting algorithm was able to cluster the microtubule disruptors, and meanwhile showed a negative correlation between paclitaxel and the other treatments. The FFT approach was able to group the samples as efficiently as checking by eye. This methodology could easily scale to group a large amount of data without visual supervision.

## 1. Introduction

Over the last 40 years, natural products (NPs) have been a major source of therapeutic compounds. Hence, 49.2% of FDA-approved drugs filed between January 1981 and September 2019 are NPs, NP derivatives, synthetic drugs based on NP pharmacophores or mimics of NPs. In the particular case of cancer, the figure increases up to 64.9% [1]. NPs and/or their derivatives continue to play a highly significant role in the drug discovery and development process [2]. It is of note that NPs can modulate the cancer microenvironment, trigger diverse cell death mechanisms (apoptosis or autophagy) and act on relevant cell signaling pathways, such as Notch, Wnt and Hedgehog [3]. NPs show a remarkable chemical diversity, but, more importantly, they have been selected through evolution for optimal interactions with biological macromolecules. These features give NPs an inherent drug-likeness and make them an ideal source of core scaffolds and capping fragments for the design and synthesis of combinatorial libraries targeted at various receptors [4].

In the drug discovery programs, NPs are tested against macromolecules of interest in molecularly-targeted assays (Targeted Drug Discovery, TDD) or against cells, isolated tissues or organs, or animals in phenotypic assays (Phenotypic Drug Discovery, PDD). A comparative analysis of both of the approaches shows that phenotypic screening prevailed in identifying first-in-class drugs, while target-based screening returned more best-in-class drugs [5]. High throughput screening (HTS) programs have allowed the rapid study of a multitude of compounds. In particular, cell-based assays for cancer research stands out. The scientific literature reveals several examples of non-mammalian and mammalian cell-based assays used for the pharmacological characterization of NPs [6]. Undoubtedly, the best known HTS program is the US National Cancer Institute (NCI) Program for Natural Product Discovery [7,8].

The challenge in a PDD strategy is to fully understand and elucidate the mode of action; in addition to the resolution of the mechanism of action (MoA), identifying the molecular target(s) affected by the molecule and responsible for its pharmacological activity. In fact, the major limitation is that in most of the studies it is only possible to depict the mode of action [9]. This complexity reduces substantially with the development of advanced testing technologies and bioinformatics tools that make PDD an accessible and realistic strategy. However, one of the main drawbacks attributed to the phenotypic strategy is that the identification of the relevant biological target(s) is relatively slow.

Cell imaging is an emerging technique to study the MoA in drug discovery programs [10,11]. The image-based profiling has become one of the most prolific methods for identifying the differences in cellular responses to treatment. The availability of automated microscopes has increased the time-cost-effectiveness ratio, collecting high content data without time-consuming experiments. The ability to observe the heterogeneous subpopulations in the same sample through single-cell resolution allows for a better understanding of the biological diversity, shadowed in the so-called big picture assays [12,13]. One of the main difficulties in the development of HTS models is the selection of a dose and assay endpoint. The correct choice of these parameters is a crucial step for the comparison of different compounds. Live imaging allows for the capture of phenotypic changes through multiple time points, reducing the bias induced by the dose selection or exposition to the compound. In this particular context, the biological profiling via Cell Painting is capable of systematically extracting and quantifying cellular morphological features [14]. In this method, the cells after drug treatment are stained with six non-antibody-based dyes that label seven organelles and cellular compartments. Then, the cells are imaged with an automated, high throughput fluorescent microscope across five channels. Cell painting only allows the phenotypic profiling at the end of the experiment. As a limitation, it is not capable of following the phenotypic changes during time, i.e., in a live imaging fashion, due to the confounding effects produced by the inherent photobleaching of the dyes or their cell phototoxicity. Recently, digital holotomography (DHT) demonstrated label-free quantitative three-dimensional (3D) imaging of cells [15]. Moreover, contrary to the fluorescence confocal microscopy, the DHT enables non-destructive, label-free 3D quantitative phase imaging.

The data management resulting from the aforementioned methodologies is often unmanageable, due to the size [16]. Therefore, the computational tools have become essential for the analysis of high content screenings. The development of artificial intelligence (AI) algorithms that characterize the bioactive compounds in order to elucidate its MoA has centered the efforts of multiple researchers in recent years [9,16,17,18]. The clustering of different compounds based on their phenotypic profiles is possible using the nearest reference approach, where each sample is classified based on its biological target or MoA [16,19].

Another limiting factor in the development of the HTS models is the proper selection of the drug dose and assay endpoint. The correct choice of these parameters is a crucial step for the comparison of the phenotypic profiles induced by the different compounds under testing. Importantly, live imaging allows for the capture of the phenotypic changes through multiple time points, reducing the bias induced by the dose selection or exposure to the drugs.

Herein, we report our preliminary results on the development of a machine learning methodology based on holotomographic cell images as a tool for the early phenotypic profiling of natural product small molecules with anticancer potential. To develop the model, we selected the non-small cell lung cancer (NSCLC) cell line SW1573, which was exposed to the widely known antimitotic drugs paclitaxel (PTX), colchicine (CLC) and vinblastine (VBL).

## 2. Results

### 2.1. Continuous Live Imaging

**Figure 1:**
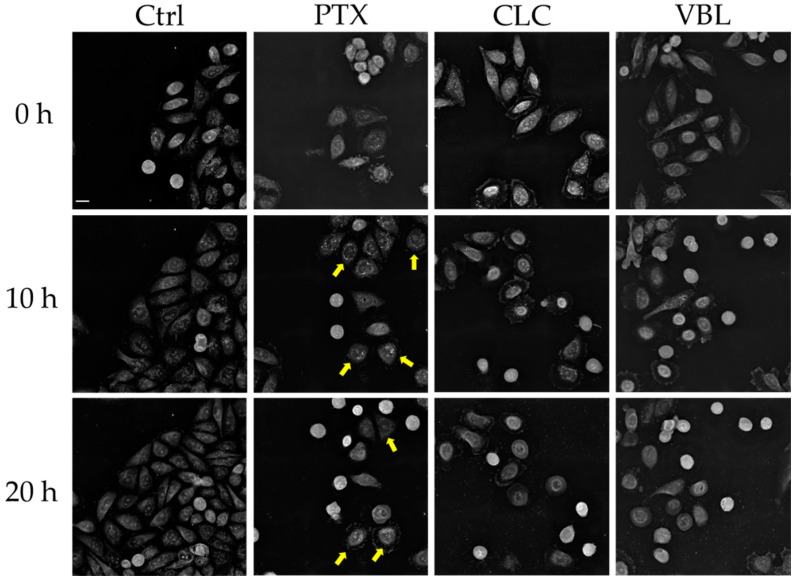
Snapshot (3D holotomography) of SW1573 cells untreated (control) and treated with PTX, CLC and VBL at 0, 10 and 20 h after exposure. Yellow arrows: subpopulation of cells showing a rounded morphology. Scale bar = 20 µm.

Due to the enhanced proliferative activity of the cancer cells, targeting cell division with small molecules has been a therapeutic strategy deeply explored for the past few decades. Among others, microtubules have emerged as the most successful targets in the treatment of neoplastic malignant diseases [20]. The microtubule-directed drugs divide into two broad groups: (a) microtubule-disrupting agents, such as CLC or VBL; and (b) microtubule stabilizers, such as taxane derivatives (PTX). However, these different modes of action are concentration dependent, with blurred limits when using the doses at a low nanomolar scale [21]. To avoid this, an equal high concentration (1 µM) of PTX, CLC and VBL was administrated to the SW1573 cells, following and visualizing every three minutes the effects over 20 h after drug exposure (Figure 1).

**Figure 2:**
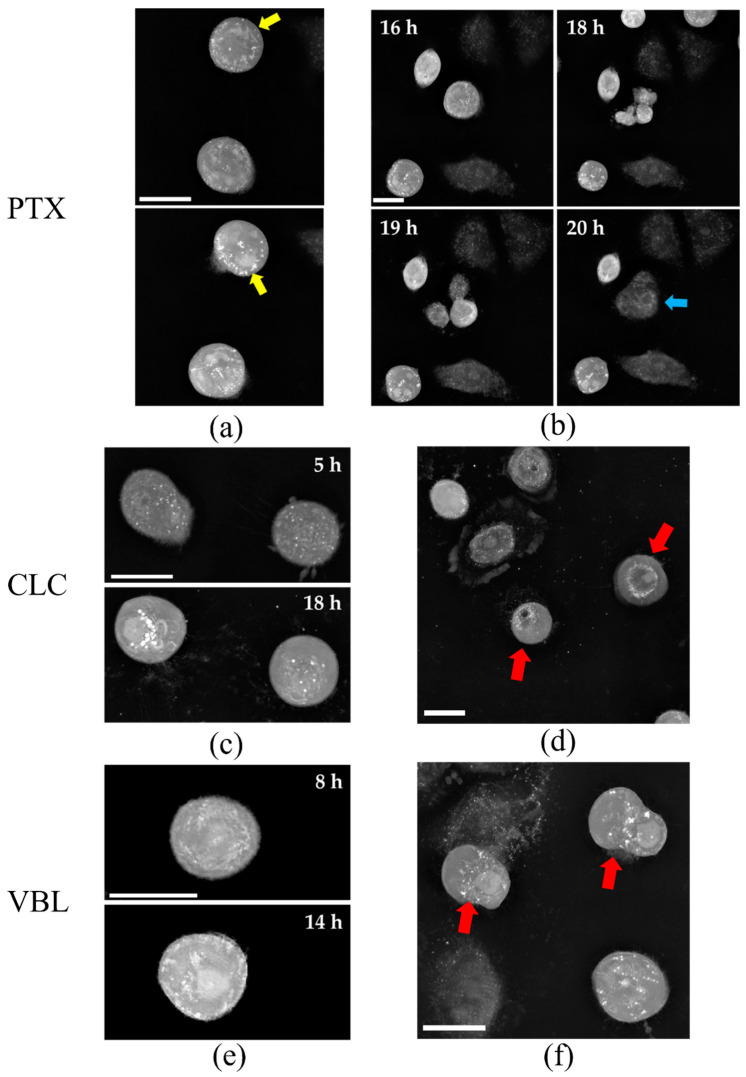
Snapshot of SW1573 cells treated with microtubule targeting drugs PTX, CLC and VBL. (**a**) Displaced mitotic spindle from the center of the cell (yellow arrows) during division, with loss of cell polarity; (**b**) Cells trying to undergo mitosis: Unresolved cytokinesis resulted in a new, polynucleated entity (blue arrow); (**c**,**e**) Mitotic cells showing disruption of cell structure, typical of microtubule destabilization; (**d**,**f**) Necrotic cells after exposure to drugs. Red arrows point to membrane breakdown, sign of necrotic cells. Scale bar = 20 µm.

PTX binds to tubulin and avoids depolymerization through microtubule stabilization. This induces mitotic arrest, thus, blocking the cells in the G_2_/M phase without the formation of a centered mitotic spindle [22]. This feature can be easily observed in the live cell imaging experiments with SW1573 cells (Figure 1). After 10 h of PTX exposure, the cells entered mitosis. In contrast to the control cells, the PTX-treated cells were not able to progress into mitosis. The PTX treatment produced cell subpopulations with unresolved mitosis (Figure 2a and Video S2, Supplementary Materials) but also aberrant cytokinetic cells (Figure 2b and Video S2, Supplementary Materials). The heterogenicity in the response of the cells to the drug happens due to the different stages of the cell cycle in the sample, since the population is not synchronized. The observed phenotypic effects represent the typical features of a high dose of PTX [23]. In addition, PTX-treated cells showed a more rounded morphology, with a loss of cell polarity (Figure 1 and Figure 2).

Although perturbing the normal mitotic progression through the microtubule dynamics, CLC and VBL act differently to PTX. Both of the alkaloids bind to specific β-tubulin-binding sites, resulting in the disruption of the microtubule formation and blockage of the cell cycle [24]. The exposure to CLC showed an inhibition of the microtubule polymerization, resulting in cell structure disruption, with morphological effects besides the cell cycle blockage. The microscope images depicted these features, showing a different response to treatment than PTX (Figure 1), with marked differences in the cellular shape. At early time points (2 h), the cell area is clearly decreased, with a rounded and diminished nucleus area surrounded by a contracted cytoplasm. This phenotype could be a consequence of microtubule disruptions, as previously reported [25]. The number of cells entering mitosis is reduced when compared to the control and to the PTX-treated samples, ending as necrotic structures in shorter time periods (Figure 1). The other cells seemed to enter mitosis, but the mitotic spindle never formed, and there was no progression into the cell cycle with the absence of recognizable division structures (Figure 2c,e and Video S3, Supplementary Materials).

Similarly to CLC, the vinca alkaloid VBL depolymerized the microtubules of SW1573 cells at the tested dose. The mode of action resembled that of CLC, i.e., most of the phenotypical effects were common to both drugs. The exposure to VBL induced a nuclear reduction, cytoplasm contraction and an overall cell area reduction, as well as the absence of mitotic structures (Figure 1). Moreover, cell death by necrosis occurred at similar time points to those observed for the CLC-treated cells (Figure 2d,f; Videos S3 and S4, Supplementary Materials).

### 2.2. Phenotypic Parameters

**Figure 3:**
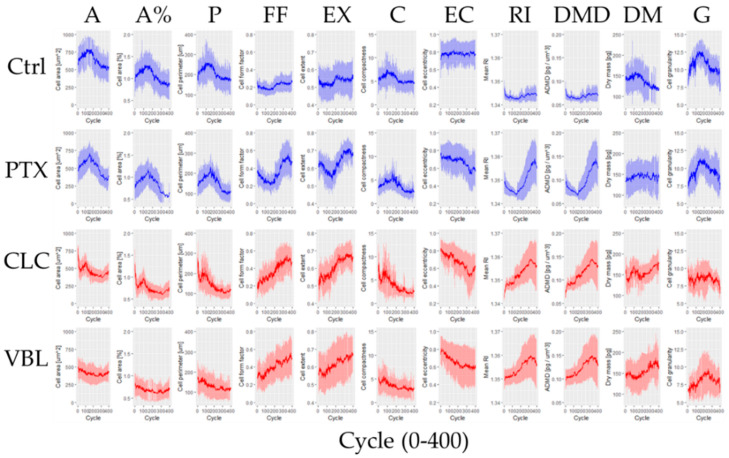
Time series of the 11 phenotypic-related parameters (mean and standard deviation): (A) Cell area (0–1000 µm^2^); (A%) Cell area (0.4–2%); (P) Cell perimeter (50–400 µm); (FF) Cell form factor (0–0.8); (EX) Cell extent (0.4–0.8); (C) Cell compactness (0–15); (EC) Cell eccentricity (0.2–1); (RI) Mean RI (1.34–1.37); (DMD) Average dry mass density (0.05–0.2 pg/µm^3^); (DM) Dry mass (80–250 pg); and (G) Cell granularity (5–15). For larger individual graphs please refer to Table S1 (Supplementary Materials). For each phenotypic parameter graph, dark lines represent mean values and shaded areas denote standard deviation.

After segmentation of the single cells over the images of the 400 cycles, the analysis inferred from the refractive index (RI) measurements for each individual cell afforded the corresponding values for the 11 phenotypic-related parameters (Table 1) at each time point (3 min for 20 h, i.e., 400 cycles). Thus, a set of 11 time-dependent graphs were obtained for each sample that could allow the inference of some differential modes of action (Figure 3). For example, the *Average Dry Mass Density* (DMD) showed a different pattern depending on the mode of action of the drugs under evaluation (Figure 3, DMD). In agreement with the observed images, CLC and VBL showed the same curve shape, with a positive slope along the cycles and a slight fall at the end. In contrast, PTX differed from the depolymerizing drugs with a clear decrease during almost the entire first half of the cycles. As expected, the control cells differed from all of the drug treatments. By definition, DMD depends on *Dry Mass* (DM) and cell volume. As observed, the DM changes are negligible (Figure 3, DM), suggesting that the increased DMD corresponds to a decrease in cell volume, as previously depicted. The more pronounced slope of DMD in the PTX treatment could result in a number of cells entering into a blocked division fate, showing a more circular phenotype. A similar result happens with cell area changes (Figure 3, A and A%).

### 2.3. Fast Fourier Transform Analysis of Phenotypic Parameters

A visual inspection of the time series from the life cell-imaging results (Figure 3) allows the determination of the qualitative similarity between VBL and CLC, with respect to the non-treated and PTX-treated cells (highlighted with different colors). These differences could be explained on the basis of the molecular mechanisms of the compounds. The interpretation and analysis of the 11 phenotypic parameters in this manner is laborious and time consuming. Certainly, the analysis is unrealistic for the implementation of HTS when using 96-well plates. Therefore, we envisioned a combination of holotomography with AI to automate the data handling.

**Figure 4:**
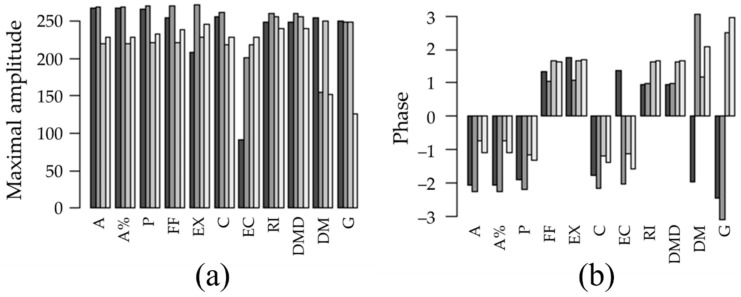
FFT analysis of the time series of the 11 phenotypic related parameters. Absolute values of (**a**) maximal amplitude and (**b**) phase. (A) Cell area (µm^2^); (A%) Cell area (%); (P) Cell perimeter (µm); (FF) Cell form factor; (EX) Cell extent; (C) Cell compactness; (EC) Cell eccentricity; (RI) Mean RI; (DMD) Average dry mass density (pg/µm^3^); (DM) Dry mass (pg); and (G) Cell granularity. Column bar groups correspond, from left to right, to control cells, PTX, CLC and VBL treatment.

Using the imaging system exclusively as a source of data, we developed an algorithm that could cluster compounds with a similar mode of action. We applied the FFT to each of the time series of the 11 phenotypic parameters (Figure 3). The FFT analysis integrates the qualitative behavior of the time series into two measures, the maximal amplitude and the corresponding phase from each parameter. The absolute values of the FFT analysis are shown in Figure 4. From the data, we can observe differences for some phenotypic parameters. Although the destination of the data is the analysis by clustering rather than by eye-check, we find it noteworthy to mention the most notorious differences. With respect to cell morphology, the largest difference (both in amplitude and phase) when compared to the control cells was obtained for the cell eccentricity (Figure 4, EC). In terms of cell composition, the dry mass (Figure 4, DM) provided the most pronounced difference between the control and treated cells. More importantly, the FFT analysis provides a quantitative measure of the eye-check differences observed in the time series plots (Figure 3).

In order to run the cluster analysis, the maximal amplitudes and the phases (Figure 4) had to be scaled and centered. The normalized values for each phenotypic parameter are included in Table 2.

**Figure 5:**
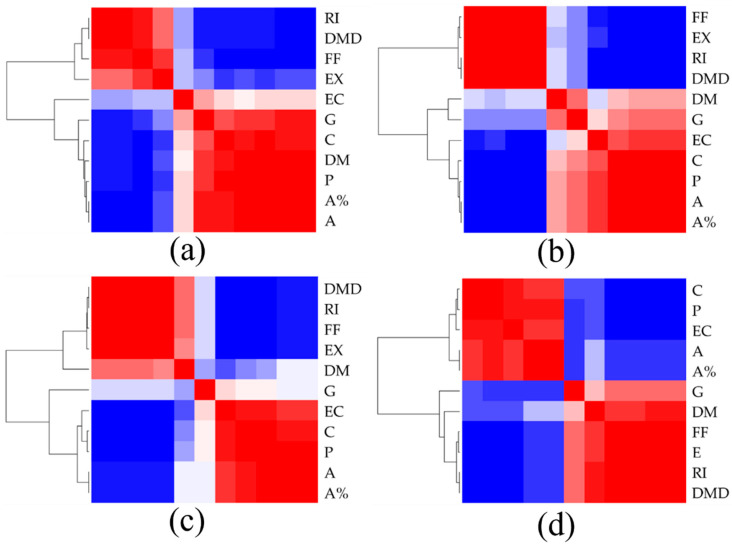
Correlation matrix (heat-map) of phenotypic parameters for (**a**) untreated SW1573 cells, and SW1573 cells treated with (**b**) PTX; (**c**) CLC; and (**d**) VBL. Legend: (A) Cell area (µm^2^); (A%) Cell area (%); (P) Cell perimeter (µm); (FF) Cell form factor; (EX) Cell extent; (C) Cell compactness; (EC) Cell eccentricity; (RI) Mean RI; (DMD) Average dry mass density (pg/µm^3^); (DM) Dry mass (pg); and (G) Cell granularity.

Figure 5 displays the correlation matrix (heat map) of the phenotypic-related parameters measured for each of the four conditions (i.e., Ctrl, PTX, CLC and VBL). Every condition displays a dissimilar correlation pattern of the 11 phenotypic parameters. This is an interesting outcome, since the phenotypic effects observed in the SW1573 cells treated with CLC (Video S3, Supplementary Materials) and those exposed to VBL (Video S4, Supplementary Materials) were very similar. Thus, the clustering of the phenotypic-related parameters seems to discriminate the subtle differences between both of the treatments.

**Figure 6:**
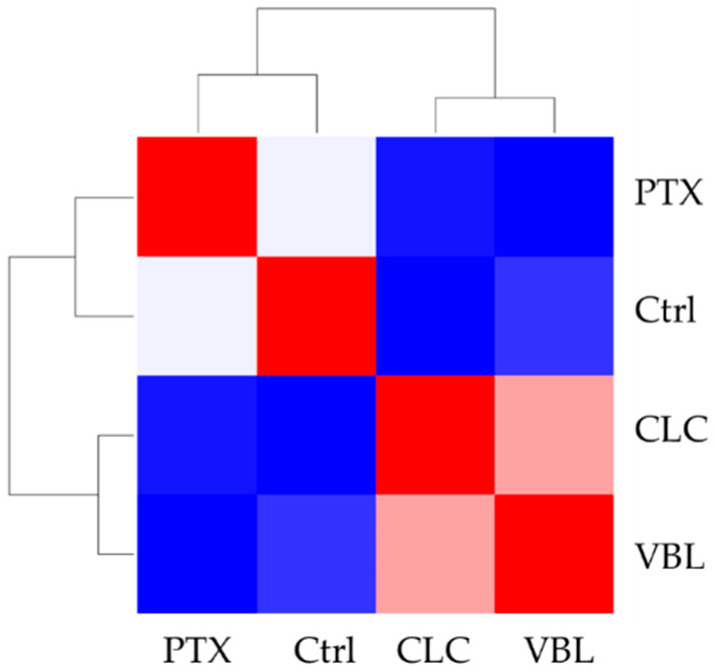
Correlation matrix (heat-map) of phenotypic parameters (Table 2) together with the cluster analysis grouping the tested conditions. Red: positive correlation, White: no correlation, Blue: negative correlation.

Figure 6 displays the correlation matrix of the values in Table 2, together with the cluster analysis grouping the four conditions. The analysis groups the untreated cells with the cells exposed to PTX, and the other cells treated with VBL and CLC are grouped. This result correlates with the visual inspection of the time series (matched colors in Figure 6). Altogether, these results show that the cluster analysis of the FFT of the time series of life cell imaging data could help with the computer-assisted phenotype annotation of the mode of action of compounds.

## 3. Discussion

Nature represents a unique source for new compounds and molecular scaffolds with potential therapeutic application. The major hurdle in these types of tests is to identify the MoA of the compounds [9]. Traditionally, the NP-based approved anticancer drugs were identified with the aid of phenotypic assays. One of the main drawbacks attributed to the PDD strategy is that the identification of the relevant biological target(s) is relatively slow. Despite this, there is no established general methodology and the scientific literature on this transcendental topic is scarce and atomized [9]. The studies concerning the in silico prediction of MoA to date relied on the data obtained from costly experimental measurements. For example, a QSAR model of compounds with antiproliferative activity allows for the prediction of whether the MoA can adjust to any of the six MoAs recognized by the NCI [26]. Unfortunately, this predictive model is not valid for small molecules with a different MoA from those established. Another model proposes combining three data sources: chemical structure; biological pathways and phenotypic responses [27]. Although the model represents an advance, it has the limitation of the low statistical significance of the results. As an added difficulty, we found that a large part of the drugs binds to more than one biological target, giving a phenotype composed of many secondary molecular effects (off-target), traditionally considered undesirable. More recently, the continuous live cell imaging allows for the visualization of the population heterogeneity at a single cell level. The advent of the DHT technique opened the way to quantitative phase imaging.

In 1985, the NCI started a HTS program-at present known as NCI60-that studies the effect of small molecules against a panel of 60 tumor cell lines of human origin, representing the nine histological types of cancer that are prevalent in the US. With the experimental data obtained (inhibitory concentration of 50% or GI_50_), algorithms such as COMPARE [28] have been developed allowing the limitation of the MoA of the compound under study. The German company Oncotest, in its screening program that employs 42 cell lines, has also applied this algorithm [29]. The main limitation of the COMPARE algorithm is the high number of cell lines (>35) needed to obtain the representative data [30]. The COMPARE algorithm was followed by the development of other algorithms for less general use, but also aimed at finding relationships between the compound sensitivity and the molecular, genomic and epigenomic expression patterns. Another example of joining forces on this issue at a global and open level is the NCI-DREAM Drug Sensitivity Prediction Challenge [31]. A group of 127 researchers analyzed 44 predictive sensitivity algorithms using 53 breast cancer cell lines and 28 drugs. Analogous to COMPARE, the studies were based on the GI_50_ values. The study revealed that including previous knowledge about biological routes improved the predictions. More recently, the correlation was studied between the GI_50_ values and the results obtained in clinical trials in 482 cell lines of breast cancer patients and 280 cell lines of multiple myeloma patients [32]. The trend indicated that the next step should be integration with the genetic data [33].

In this study, we report on an AI algorithm based on the ability to cluster the phenotypic responses of tumor cells exposed to antiproliferative compounds. We envisioned the combination of RI-derived experimental metrics (Table 1) and machine learning techniques in order to establish a methodology that could aid in the early pharmacological profiling of investigational small molecules. As a model to develop the algorithm, we selected three well-known microtubule-interacting drugs. The visual analysis of the three microtubules targeting agents showed clear differences in cell behavior under the same conditions (Figure 1 and Figure 2). Interestingly, the microtubule stabilizer PTX exert a different response in the SW1573 cells than the two microtubule disruptors, CLC and VBL. PTX produced its antiproliferative effect through a mitotic blockage without a correctly formed mitotic spindle, whilst CLC and VBL showed a different mode of action, with a diminished nucleus area, cytoplasm contraction and less cells entering in mitosis, probably due to microtubule disruption. This interpretation is supported by the parameters obtained after the analysis. For instance, the ADMD and cell area estimation from the RI values fitted well with the observed outcome videos.

In our model, we explored the possibility of reducing the cell line numbers (thus, enhancing feasibility), while using meaningful phenotypic metrics with the highest biological relevance [15], i.e., changes in cell morphology and cell content (Table 1). DHT allowed the inclusion of an extra parameter into consideration-exposure time. Perhaps, this is the most powerful contribution of the continuous label-free live cell imaging. Traditionally, the bioactivity studies were conducted on limited and predefined time (end) points. In this study, the SW1573 cells were monitored every three minutes for 20 h (400 cycles), providing for each individual cell a data matrix of 11 phenotypic parameters followed over the 400 cycles (Figure 3). During this work, we found that even for a small subset of compounds, the large amount of data derived from the measurements need to be handled with the aid of machine learning. Although the number of samples assayed in this work are manageable for individual manipulation, the transition to a high throughput scale would be extremely time-consuming and unmanageable without a vast number of human resources. Therefore, the implementation of computational tools becomes necessary for an efficient scaling of these methodologies. Since the time series displayed a waveform function at different points, we decided to use the FFT to transform the time series of the parameters into their corresponding amplitudes and phases. From this frequency plot, the maximal amplitude and the corresponding phase could reproduce the coarse-grain qualitative behavior of the dynamics of the time-series data. The resulting data could serve as a sort of dimension reduction approach for the further multidimensional analysis of the data. To the best of our knowledge, this is the first time that the FFT was applied to analyze continuous live cell-imaging-derived data. We speculate that the calculation of FFT for each individual cell instead of the mean values of the population could help give the possibility of screening outliers and assessing the biological variability and the differential response to tested drugs.

The algorithm clustered the microtubule disruptors; meanwhile, it showed a negative correlation between PTX and the other two treatments (Figure 6). This is explained by the marked morphology differences between the PTX treatment and the CLC- and VBL-treated cells. These results represent a good preliminary starting point for our prediction model. The FFT approach, even when reducing all of the information from the times series into one value of amplitude and the corresponding phase, is able to group the samples as efficiently as an eye check. This methodology could easily scale to group a large amount of data without visual supervision.

For this preliminary study, we were limited to testing the microtubule-targeting agents against one specific cell line. The incorporation of more compounds that affect microtubules, as well as molecules with other modes of action, is necessary for the strengthening of the algorithm. Furthermore, tests in different cell lines would help to find more specificities in the phenotypic profiling of the small molecules. To date, the evaluation under those conditions would be a non-productive effort in the absence of a system which provides enough sensibility to identify the similarities between the samples. The preliminary model represents a valuable cornerstone for the integration of label-free continuous live cell-imaging and our analysis system into PDD programs.

## 4. Materials and Methods

### 4.1. Chemicals and Reagents

The PTX, CLC and VBL were purchased from Sigma-Aldrich (St. Louis, MO, USA). The stock solutions of the drugs were prepared in DMSO (PTX at 0.4 mM; CLC and VBL at 10 mM).

### 4.2. Cell Lines, Passage and Maintenance

The non-small cell lung cancer cell line SW1573 was used for the in vitro experiments. The cell line was kindly provided by Prof. G. J. Peters (VUMC, Amsterdam). The cells were grown in RPMI 1640 medium supplemented with 2 mM glutamine, 5% fetal bovine serum (FBS), and 100 U/mL penicillin and 0.1 mg/mL streptomycin as the antibiotics. They were incubated at 37 °C, in a humidified atmosphere 5% CO_2_ and maintained at low passage.

### 4.3. Continuous Live Cell Imaging

To evaluate the drug-induced phenotypic changes related to the different biochemical mechanisms of action, live cell microscopy was performed using CX-A label-free cell imaging system (Nanolive S.A., Switzerland). First, the SW1573 cells were trypsinized, and after counting, 100,000 cells were seeded on 35 mm high glass-bottom µ-dish from IBIDI. After 24 h, the medium was changed for RPMI 1640 without phenol red, and the cells were treated with different drugs targeting the microtubules (paclitaxel, vinblastine, and colchicine) at a concentration of 1 µM. Each treatment was repeated once, but measures were obtained for each cell in the imaging area. Immediately after that, the plates were placed in the CX-A and images from a 236 µm × 236 µm area were taken every 3 min for 20 h.

After image acquisition, the Eve segmentation and analysis software (Nanolive S.A., Switzerland) was used to evaluate the 11 phenotypic-related parameters, which were subdivided into two groups: (1) Cell morphology: Cell area (µm^2^), Cell area (%), Cell perimeter, Cell eccentricity, Cell granularity, Cell compactness, Cell form factor and Cell extent; and (2) Cell composition: Mean refraction index, Average dry mass density (pg/µm^3^) and Dry mass (pg). The measurements were obtained for each cell at all of the imaging cycles for each treatment. The initial cell amount of the field area was in the range 15–25 cells.

### 4.4. Prediction Algorithm Design

For the design of the model, we used Rstudio version 2022.02.2+485 [34]. The data directly obtained from Nanolive’s Eve analysis software (as csv type file) were recast using the function recast from package reshape 2 to obtain the time series of the phenotypic parameters for each cell. After calculating the mean at each time interval (400 cycles in total), the dataset was centered using the function scale. Then, the FFT was calculated using function fft (Equation 1), which is a mathematical transform that decomposes the functions depending on time into the functions depending on temporal frequency [35]. After this transformation, the period and phase values were obtained for every phenotypic parameter and condition. Each period and phase value would represent the qualitative behavior of the average of all of the cells. The frequency and the corresponding phase with the maximal amplitude (usually the lower frequency) informs about the main trend of the data in time, namely increase, decrease or steady. The amplitude of this frequency would represent the relative similarity to a pure wave signal and the phase informs about the part of the wave represented in the data (from bottom to top, from top to bottom). For the cluster analysis, the frequency and phase corresponding to the maximal amplitude of the FFT of the time series, for each variable, were scaled, centered and used.

(1) $$\mathrm{FFT}\left(x_{n}\right)=\overset{N-1}{\underset{n=0}{\sum }}x_{n}e^{-i2\pi \mathrm{kn}/N}\;$$

## 5. Conclusions

In summary, we explored the combination of continuous label-free live cell-imaging and AI techniques to develop an algorithm that could help in the study of the mode of antiproliferative action of NPs against human tumor cells. The novelty of our methodology focuses on two main features with the highest relevance, (a) meaningful phenotypic metrics, and (b) FFT of the times series of the phenotypic parameters into their corresponding amplitudes and phases. Further studies with other cell lines and more drugs are being carried out in 96-well plates. The results lie outside the scope of this preliminary study and will be reported elsewhere.

## Figures and Tables

**Table 1:** Phenotypic related parameters grouped by subtypes.

Subtype	Parameter	Units
Cell morphology	Cell area	µm^2^
	Cell area	%
	Cell perimeter	µm
	Cell form factor	-
	Cell extent	-
	Cell compactness	-
	Cell eccentricity	-
Cell composition	Mean RI	-
	Average dry mass density	pg/µm^3^
	Dry mass	pg
	Cell granularity	-

**Table 2:** Normalized parameters obtained after FFT analysis of the time series data.

FFT Factor ^1^	Sample	A ^2^	A%	P	FF	EX	C	EC	RI	DMD	DM	G
Maximal amplitude	Control	1.18	1.18	0.96	0.68	−0.02	0.89	−1.24	0.70	0.70	1.38	0.60
	PTX	1.22	1.22	1.03	0.93	1.02	1.00	0.62	1.05	1.05	−0.89	0.59
	CLC	0.27	0.27	0.38	0.43	0.59	0.41	1.08	0.46	0.46	−0.95	−1.29
	VBL	0.06	0.06	0.17	0.14	0.31	0.22	0.91	0.91	0.91	1.27	0.59
Phase	Control	−0.92	−0.92	−0.91	0.21	0.36	−0.87	1.28	−0.89	−0.89	−1.68	−1.03
	PTX	−1.10	−1.10	−1.20	0.04	−0.02	−1.27	−0.87	−0.80	−0.80	1.39	−1.26
	CLC	−0.06	−0.06	−0.26	0.38	0.33	−0.45	−0.58	0.46	0.46	0.80	0.86
	VBL	0.26	0.26	−0.10	0.41	0.30	−0.27	−0.30	0.42	0.42	0.24	0.71

^1^ Scaled and centered; ^2^ (A) Cell area (µm^2^); (A%) Cell area (%); (P) Cell perimeter (µm); (FF) Cell form factor; (EX) Cell extent; (C) Cell compactness; (EC) Cell eccentricity; (RI) Mean RI; (DMD) Average dry mass density (pg/µm^3^); (DM) Dry mass (pg); and (G) Cell granularity.

## Data Availability

The data presented in this study are available on request from the corresponding author. The data are not publicly available due to privacy or ethical restrictions.

## Supplementary Materials

The following supporting information can be downloaded at: https://www.mdpi.com/article/10.3390/molecules27165261/s1, Table S1: Larger image of time series for each phenotypic parameter, Video S1: Control (untreated) SW1573 cells, Video S2: PTX-treated SW1573 cells, Video S3: CLC-treated SW1573 cells, Video S4: VBL-treated SW1573 cells.

## References

[1] Newman D.J., Cragg G.M. (2020). Natural products as sources of new drugs over the nearly four decades from 01/1981 to 09/2019. J. Nat. Prod..

[2] Huang M., Lu J.-J., Ding J. (2021). Natural products in cancer therapy: Past, present and future. Nat. Prod. Bioprospecting.

[3] Hashem S., Ali T.A., Akhtar S., Nisar S., Sageena G., Ali S., Al-Mannai S., Therachiyil L., Mir R., Elfaki I. (2022). Targeting cancer signaling pathways by natural products: Exploring promising anti-cancer agents. Biomed. Pharmacother..

[4] Hong J. (2011). Role of natural product diversity in chemical biology. Curr. Opin. Chem. Biol..

[5] Swinney D.C. (2013). Phenotypic vs. target-based drug discovery for first-in-class medicines. Clin. Pharm. Ther..

[6] Wilson B.A.P., Thornburg C.C., Henrich C.J., Grkovic T., O’Keefe B.R. (2020). Creating and screening natural product libraries. Nat. Prod. Rep..

[7] Thornburg C.C., Britt J.R., Evans J.R., Akee R.K., Whitt J.A., Trinh S.K., Harris M.J., Thompson J.R., Ewing T.L., Shipley S.M. (2018). NCI program for natural product discovery: A publicly-accessible library of natural product fractions for high-throughput screening. ACS Chem. Biol..

[8] Grkovic T., Akee R.K., Thornburg C.C., Trinh S.K., Britt J.R., Harris M.J., Evans J.R., Kang U., Ensel S., Henrich C.J. (2020). National Cancer Institute (NCI) program for natural products discovery: Rapid isolation and identification of biologically active natural products from the NCI prefractionated library. ACS Chem. Biol..

[9] Trapotsi M.-A., Hosseini-Gerami L., Bender A. (2022). Computational analyses of mechanism of action (MoA): Data, methods and integration. RSC Chem. Biol..

[10] Chandrasekaran S.N., Ceulemans H., Boyd J.D., Carpenter A.E. (2020). Image-based profiling for drug discovery: Due for a machine-learning upgrade?. Nat. Rev. Drug Discov..

[11] Caicedo J.C., Singh S., Carpenter A.E. (2016). Applications in image-based profiling of perturbations. Curr. Opin. Biotechnol..

[12] Perlman Z.E., Slack M.D., Feng Y., Mitchison T.J., Wu L.F., Altschuler S.J. (2004). Multidimensional drug profiling by automated microscopy. Science.

[13] Simm J., Klambauer G., Arany A., Steijaert M., Wegner J.K., Gustin E., Chupakhin V., Chong Y.T., Vialard J., Buijnsters P. (2018). Repurposing high-throughput image assays enables biological activity prediction for drug discovery. Cell Chem. Biol..

[14] Bray M.A., Singh S., Han H., Davis C.T., Borgeson B., Hartland C., Kost-Alimova M., Gustafsdottir S.M., Gibson C.C., Carpenter A.E. (2016). Cell Painting, a high-content image-based assay for morphological profiling using multiplexed fluorescent dyes. Nat. Protoc..

[15] Park Y.K., Depeursinge C., Popescu G. (2018). Quantitative phase imaging in biomedicine. Nat. Photonics.

[16] Cox M.J., Jaensch S., Van de Waeter J., Cougnaud L., Seynaeve D., Benalla S., Koo S.J., Van Den Wyngaert I., Neefs J.M., Malkov D. (2020). Tales of 1,008 small molecules: Phenomic profiling through live-cell imaging in a panel of reporter cell lines. Sci. Rep..

[17] Wawer M.J., Li K., Gustafsdottir S.M., Ljosa V., Bodycombe N.E., Marton M.A., Sokolnicki K.L., Bray M.A., Kemp M.M., Winchester E. (2014). Toward performance-diverse small-molecule libraries for cell-based phenotypic screening using multiplexed high-dimensional profiling. Proc. Natl. Acad. Sci. USA.

[18] Kang J., Hsu C.H., Wu Q., Liu S., Coster A.D., Posner B.A., Altschuler S.J., Wu L.F. (2016). Improving drug discovery with high-content phenotypic screens by systematic selection of reporter cell lines. Nat. Biotechnol..

[19] Loo L.-H., Wu L.F., Altschuler S.J. (2006). Image-based multivariate profiling of drug responses from single cells. Nat. Methods.

[20] Jordan M.A., Wilson L. (2004). Microtubules as a target for anticancer drugs. Nat. Rev. Cancer.

[21] Yang C.P.H., Horwitz S.B. (2017). Taxol^®^: The first microtubule stabilizing agent. Int. J. Mol. Sci..

[22] Shannon K.B., Canman J.C., Moree C.B., Tirnauer J.S., Salmon E.D. (2005). Taxol-stabilized microtubules can position the cytokinetic furrow in mammalian cells. Mol. Biol. Cell.

[23] Hornick J.E., Bader J.R., Tribble E.K., Trimble K., Breunig J.S., Halpin E.S., Vaughan K.T., Hinchcliffe E.H. (2008). Live-cell analysis of mitotic spindle formation in taxol-treated cells. Cell Motil. Cytoskelet..

[24] Fanale D., Bronte G., Passiglia F., Calò V., Castiglia M., Piazza F.D., Barraco N., Cangemi A., Catarella M.T., Insalaco L. (2015). Stabilizing versus destabilizing the microtubules: A double-edge sword for an effective cancer treatment option?. Anal. Cell. Pathol..

[25] Weber A., Iturri J., Benitez R., Zemljic-Jokhadar S., Toca-Herrera J.L. (2019). Microtubule disruption changes endothelial cell mechanics and adhesion. Sci. Rep..

[26] Whitebay E.A., Gasem K.A.M., Neely B.J., Ramsey J.D. (2013). In silico prediction of mechanism of action for cancer therapeutics. Mol. Inf..

[27] Liggi S., Drakakis G., Koutsoukas A., Cortes-Ciriano I., Martínez-Alonso P., Malliavin T.E., Velazquez-Campoy A., Brewerton S.C., Bodkin M.J., Evans D.A. (2014). Extending in silico mechanism-of-action analysis by annotating targets with pathways: Application to cellular cytotoxicity readouts. Future Med. Chem..

[28] Paull K.D., Shoemaker R.H., Hodes L., Monks A., Scudiero D.A., Rubinstein L., Plowman J., Boyd M.R. (1989). Display and analysis of patterns of differential activity of drugs against human tumor cell lines: Development of mean graph and COMPARE algorithm. J. Natl. Cancer Inst..

[29] Telle W., Kelter G., Fiebig H.-H., Jones P.G., Lindel T. (2014). Total synthesis and cytotoxicity of the marine natural product malevamide D and a photoreactive analog. Beilstein J. Org. Chem..

[30] Reinhold W.C., Sunshine M., Liu H., Varma S., Kohn K.W., Morris J., Doroshow J., Pommier Y. (2012). CellMiner: A web-based suite of genomic and pharmacologic tools to explore transcript and drug patterns in the NCI-60 cell line set. Cancer Res..

[31] Costello J.C., Heiser L.M., Georgii E., Gönen M., Menden M.P., Wang N.J., Bansal M., Ammad-ud-din M., Hintsanen P., Khan S.A. (2014). A community effort to assess and improve drug sensitivity prediction algorithms. Nat. Biotechnol..

[32] Turki T., Wei Z. (2017). A link prediction approach to cancer drug sensitivity prediction. BMC Syst. Biol..

[33] Johannessen C.M., Clemons P.A., Wagner B.K. (2015). Integrating phenotypic small-molecule profiling and human genetics: The next phase in drug discovery. Trends Genet..

[34] RStudio Team (2022). RStudio: Integrated Development Environment for R.

[35] Wang S. (2017). Applications of Fourier transform to imaging analysis. J. R. Stat. Soc..

