# Supplementary material for: Early Pharmacological Profiling of Antiproliferative Compounds by Live Cell Imaging
Source: Molecules. 2022 Aug 17;27(16):5261. doi: 10.3390/molecules27165261 (PMC9415461; doi:10.3390/molecules27165261)
Supplement: Supplementary file 1 [file molecules-27-05261-s001.zip › molecules-1834059-supplementary.pdf]

Early Pharmacological Profiling of Antiproliferative Compounds by Live Cell Imaging

Adrián Puerta [1], Aday González-Bakker [1], Guido Santos [2] and José M. Padrón [1,*]

[1] BioLab, Instituto Universitario de Bio-Orgánica "Antonio González", Universidad de La Laguna, Avenida Astrofísico Francisco Sánchez 2, 38206 La Laguna, Spain;

[2] Departament of Biochemistry, Microbiology, Cell Biology and Genetics, Faculty of Sciences, Universidad de La Laguna, Avenida Astrofísico Francisco Sánchez s/n, 38206 La Laguna, Spain;

* Correspondence: jmpadron@ull.es; Tel.: +34 922 316 502 ext. 6126

CONTENTS

Table S1. Time series plots 2

Table S1. Time series plots of the 11 phenotypic related parameters.[a]

Ctrl	PTX	CLC	VBL
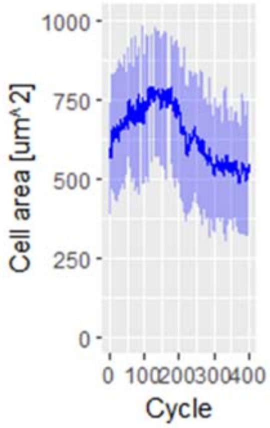	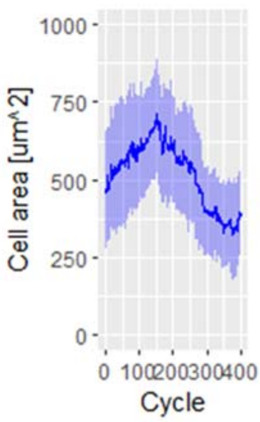	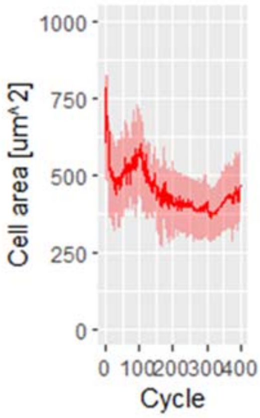	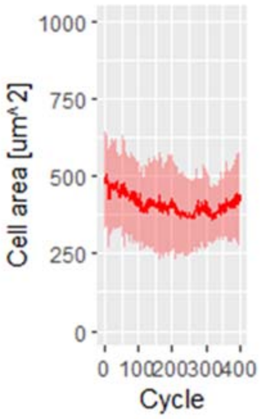
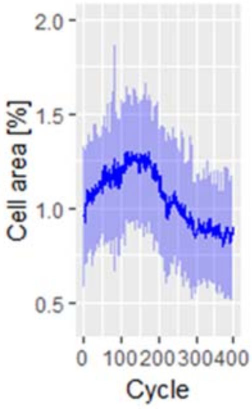	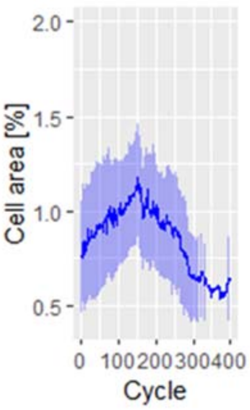	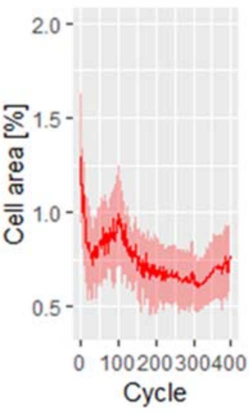	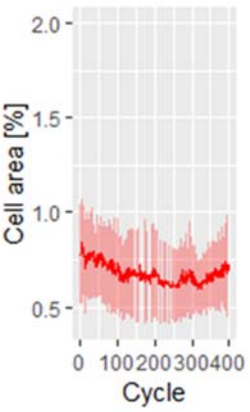
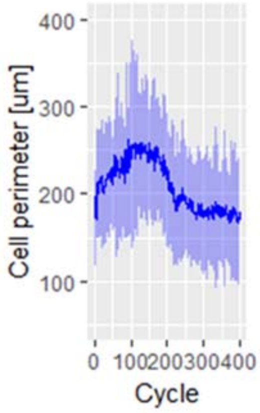	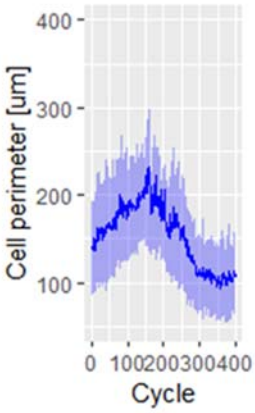	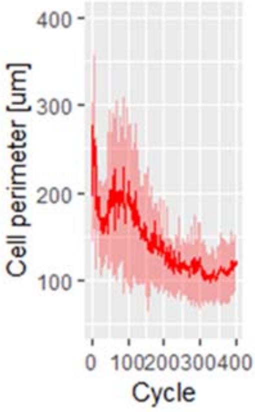	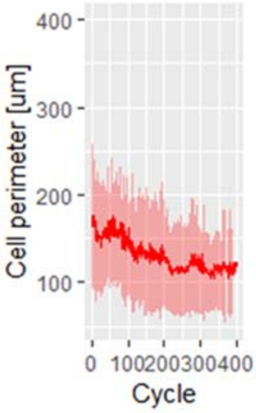
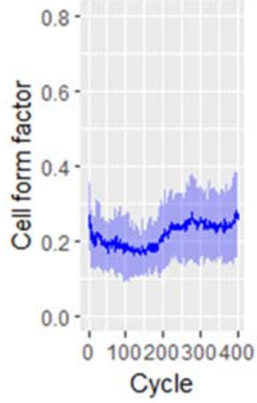	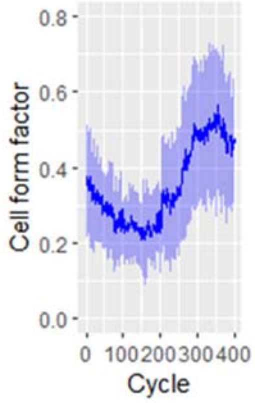	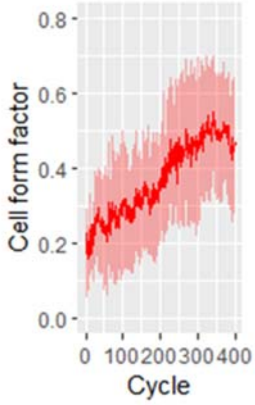	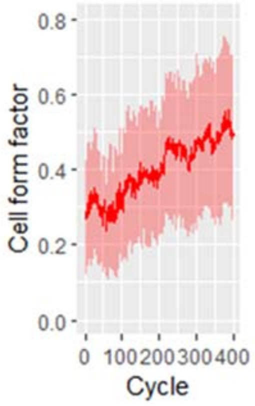

Table S1. *(continued)*

Ctrl	PTX	CLC	VBL
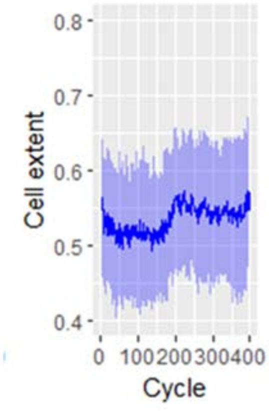	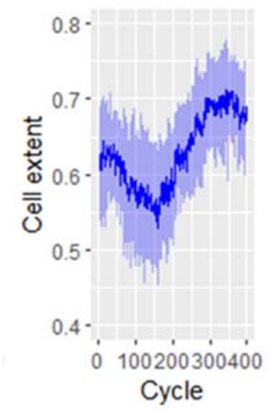	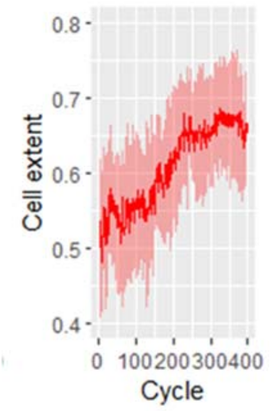	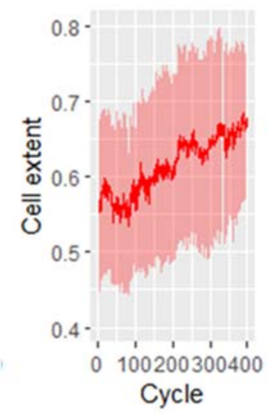
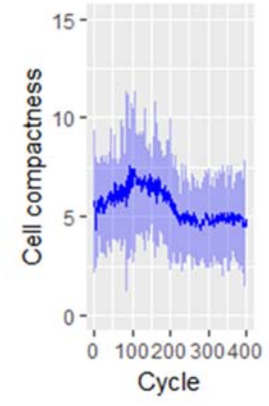	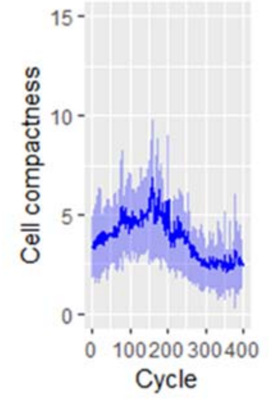	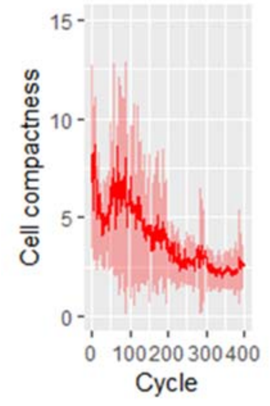	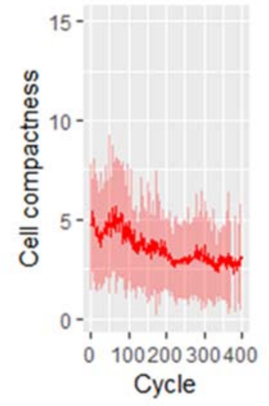
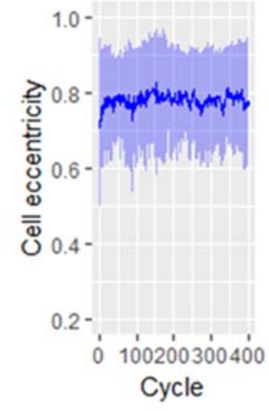	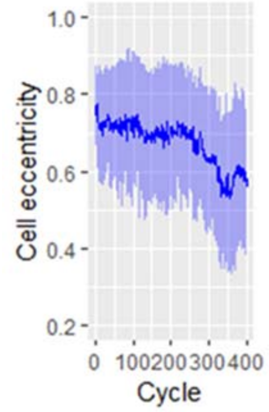	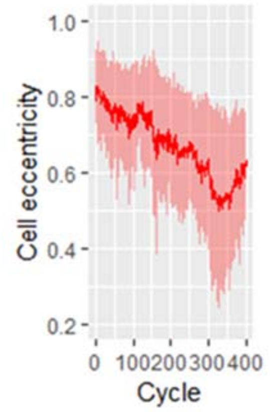	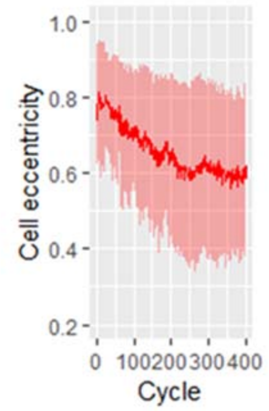
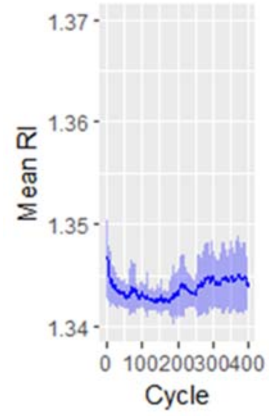	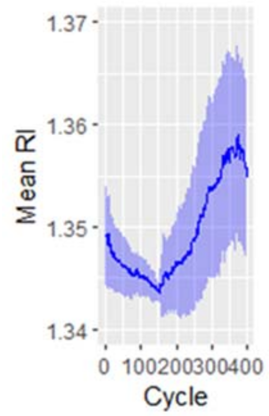	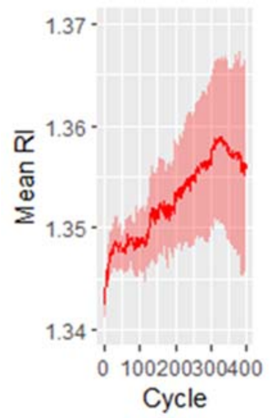	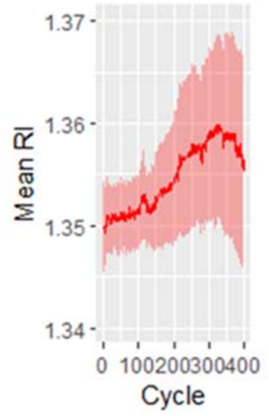

Table S1. *(continued)*

Ctrl	PTX	CLC	VBL
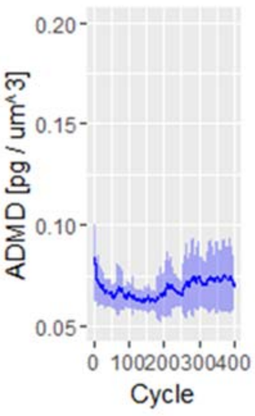	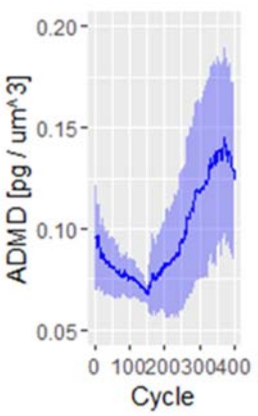	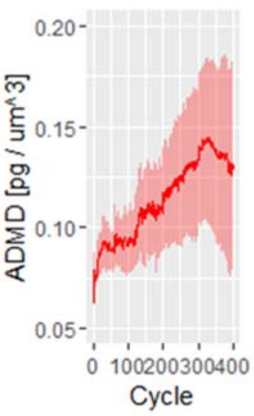	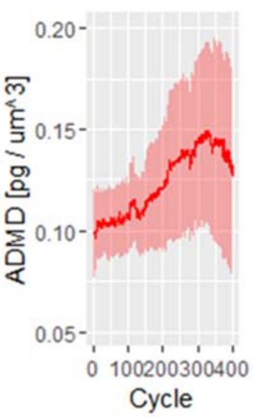
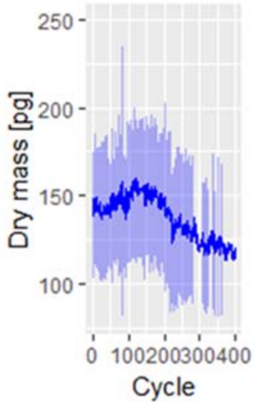	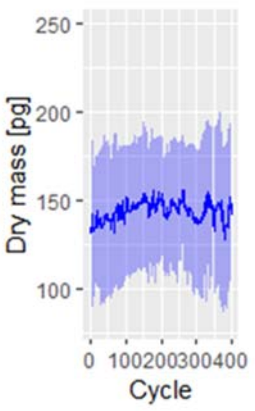	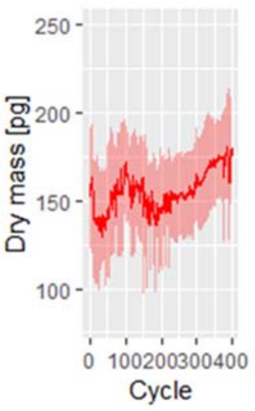	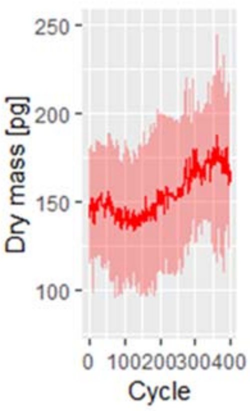
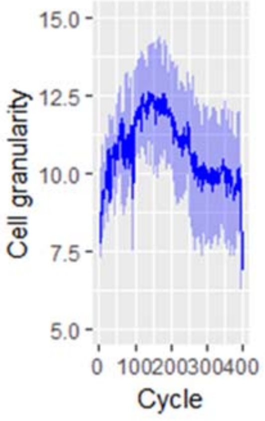	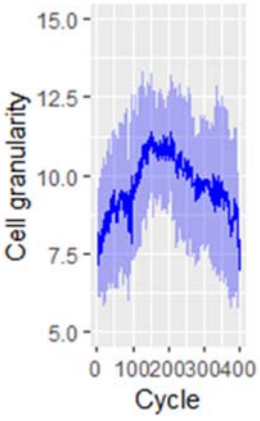	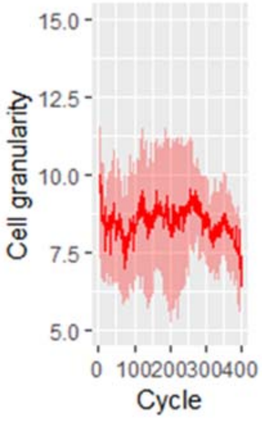	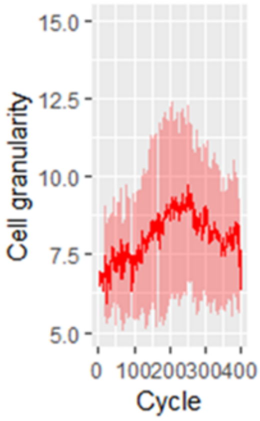

[a] For each phenotypic parameter graph, dark lines represent mean values and shaded areas denote standard deviation.
